# Design, synthesis, and density functional theory studies of a new selective chemosensor for Pb^2+^

**DOI:** 10.1016/j.heliyon.2023.e20206

**Published:** 2023-09-15

**Authors:** Hamid Hadi, Gassoumi Bouzid, Samia Nasr, Houcine Ghalla, Rafik Ben Chaabane, Sahbi Ayachi

**Affiliations:** a Department of Chemistry, Physical Chemistry group, Lorestan University, Khorramabad, Iran; b Laboratory of Advanced Materials and Interfaces (LIMA), Faculty of Sciences, University of Monastir, 5019 Monastir, Tunisia; c Research Center for Advanced Materials Science (RCAMS), King Khalid University, P.O. Box 9004, Abha 61413, Saudi Arabia; d Chemistry Department, Faculty of Science, King Khalid University, P.O. Box 9004, Abha 61413, Saudi Arabia; e Quantum and Statistical Physics Laboratory, Faculty of Sciences, University of Monastir, 5019 Monastir, Tunisia; f Laboratory of Physico-Chemistry of Materials (LR01ES19), Faculty of Sciences, University of Monastir, 5019 Monastir, Tunisia

**Keywords:** Colorimetric, Chemosensor, NMR, UV–Vis, DFT, QTAIM

## Abstract

Herein, we have focused on a new colorimetric ligand synthesized from the reaction of 2-hydroxy-5-methylbenzene-1,3-dialdehyde with 2-amino-thiophenol, and investigated its activity as a sensor. In this regard, the sensory activity of the ligand towards different ions (Mn^2+^, Cu^2+^, Co^2+^, Fe^2+^, Fe^3+^, Zn^2+^, Ni^2+^, Cd^2+^, Ag^+^, Na^+^, Cs^+^, Mg^2+^, Al^3+^, Ba^2+^, K^+^, and Pb^2+^) was studied. The specificity of ion bindings is discussed through UV–Vis analysis. The ligand that was synthesized showed remarkable sensitivity, with a detection limit of 0.001 ppb. Additionally, the presence of Pb^2+^ ions can be visually detected through a color change from colorless to yellow. In the last part of this work, we seek to predict the available experimental measurements. Density functional theory (DFT) and quantum theory of atoms in molecules (QTAIM) are employed to examine the bonding between the ligand and the Pb^2+^ ion. The effect of water solvent was thoroughly examined for all the steps via the conductor-like Polarizable Continuum Model (CPCM). The theoretical findings revealed that electronic properties, including energy gap, adsorption energy, charge/energy transfer, and optical characteristics, undergo significant changes when Pb^2+^ cations are present. Hence, it can be inferred that the newly synthesized chemosensor (NC) is highly efficient in detecting Pb^2+^.

## 1 Introduction

Lead (Pb^2+^) is a heavy toxic metal that has no useful physiological role in the human body [[1], [2], [3]]. Since Pb^2+^ ions are not degradable, exposure to lead has adverse consequences for human health [[4], [5], [6]]. For this reason, the detection of lead in the environment (due to the wide use of lead metal in various industries such as automotive, oil, and paint) has become a global concern today [[7], [8], [9]]. Drinking water is one possible source of lead exposure. Since then, the World Health Organization (WHO) has given a guideline value of 10 μg/L for Pb^2+^ in drinking water [10]. Considering the adverse effects of Pb^2+^ on human health, it seems necessary to use simple, sensitive, and reliable methods to detect this element in different environments. There are various methods, such as colorimetric, UV–Vis and fluorescence optical responses, and electrochemical, to identify Pb^2+^ ions [[11], [12], [13], [14]]. Although these methods have shown good performance in the determination of Pb^2+^, they require a controlled laboratory environment, a long operation time, and pure chemicals. In order to solve these problems, electrochemical sensors or molecular probes were designed for specific purposes with the help of the theoretical branch of host-guest supramolecular chemistry [15,16].

The idea of being able to reuse chemosensors makes them an appealing option for chemical detection, in contrast to current methods of instrumental detection. They are potentially more effective, economical, and reliable detectors of targets such as drugs, ions, and biological molecules. Molecular sensors of the host-guest type are particularly advantageous as they are economical and mobile, comparable to most instruments in terms of sensitivity and detection limits. These characteristics make molecular sensors suitable for numerous applications that require on-site detection. In many cases, samples cannot be prepared for instrumental analysis without altering their composition and many instruments are not designed for mobility, which is crucial for conducting tests in real-time.

Chemosensors are essentially host molecules that can generate a signal when a guest is attached to the host. They initially developed as macrocyclic hosts targeting cationic guests. In the meantime, however, chemosensors have now been extended to include anions and small guest molecules. They also incorporate ligands, which are non-cyclic molecules, molecular cavities, and a wide range of other categories of molecular hosts [17,18].

In the past few years, various experimental techniques have been used to produce novel chemical sensors. These sensors have been designed to selectively and efficiently capture and concentrate various types of heavy metal cations [[19], [20], [21], [22], [23], [24], [25], [26]].

The sensors of toxic cations have become a major subject nowadays. For this reason, we are interested in a new chemical sensor for the Pb^2+^ cation. Among the electrochemical sensors, colorimetric sensors have expanded due to their ability to detect the analytic with the naked eye (without the need for expensive devices). Colorimetric sensors, upon binding to a specific analytic, produce a distinct signal that is significant in terms of color change and change in absorption or emission. In the colorimetric detection of ions, various structures can act as acceptor units and selectively bind to the analytic. Schiff base compounds are an important class of color-sensitive chemical sensors that can selectively detect target ions [27,28]. The main focus of this work is the use of host-guest chemistry and colorimetric sensor design to target specific ions (Pb^2+^). In addition, the proposal of a material that is composed of a group that plays a donor (D)-acceptor (A) role based on sulfur at the same time is a gain for our detection applications. In this research, a new Schiff base colorimetric sensor was prepared from the reaction of 2-hydroxy-5-methylbenzene-1,3-dialdehyde with 2-aminothiophenol. This compound (containing a C=N dye unit with an OH-donor unit) is designed and prepared as a selective sensor for Pb^2+^ detection (by colorimetric method). Based on computational studies (DFT and QTAIM theory), the structural and electronic properties of the synthesized ligand are investigated. The electron charge transfer is deeply interpreted. Considering its low cost, easy synthesis, excellent chemical stability, and selectivity for Pb^2+^, our compound will be the most suitable for future sensor applications.

## 2 Experimental section

### 2.1 Synthesis approach

**Fig. 1:**
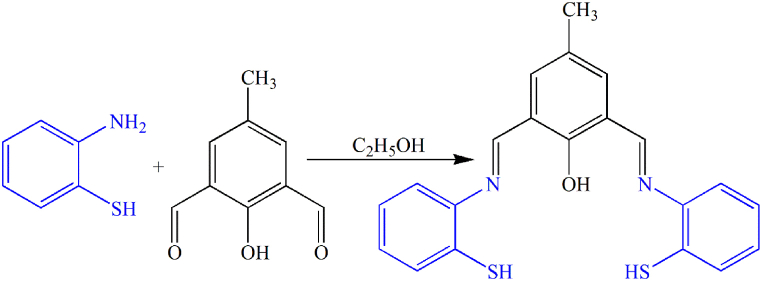
Ligand (Chemosensor) synthesis.

0.5 mmol of 2-hydroxy-5-methylbenzene-1,3-dialdehyde was dissolved with 1.1 mmol of 2-aminothiophenol in 100 mL of ethanol. Then 2–3 drops of acetic acid (as a catalyst) were added to the solution. The resulting solution was refluxed for 72 h. Then the solution is removed from the reflux and placed in a rotary oven to evaporate the solvent. After that, the precipitate was collected with filter paper. Then, the synthesized sample was purified again using acetonitrile solvent and subjected to a rotary operator (Fig. 1).

### 2.2 Sample preparation

In order to prepare a 4 × 10^−5^ M solution of the synthesized ligand, 8.6 mg of it was added to the volume in a 5 ml volumetric flask with N,N dimethylformamide. The solution of metal ions Pb(NO_3_)_2_, MnCl_2_.4H_2_O, Cu(NO_3_)_2_.3H_2_O, Co(NO_3_)_2_.6H_2_O, FeCl_3_.6H_2_O, FeCl_2_.4H_2_O, Zn(NO_3_)_2_.6H_2_O, Ni(NO_3_)_2_.6H_2_O, Cd(NO_3_)_2_.4H_2_O, AgNO_3_, NaClO_4_.H_2_O, Cs(NO_3_), Mg(NO_3_)_2_.4H_2_O, AlCl_3_.6H_2_O, Ba(NO_3_)_2_, KClO_3_ and Hg_2_Cl_2_ were prepared after the nitrate salts dissipated in distilled water. In order to determine the selectivity of the synthesized ligand, 0.2 ml of the ligand solution was added to the 17-volumetric flask of 10 ml and made up to volume with an ethanol-water solution (v/v, 90,10). Then, we added to the solution containing the ligand 10 equivalents of the aqueous solution of each of the cations. The identification of each of the cations was done in the first step using the naked eye. In the next step, the absorption intensity and colorimetric response of the ligand are measured using an UV–Vis spectrometer. A solution with a constant concentration (4 × 10^−5^ M) of the ligand was prepared and titrated with a ratio of 0–5 equivalents of the target cation. This was accomplished after evaluating the effect of the concentration on the ligand's ability to selectively detect Pb^2+^ cations.

### 2.3 Materials and spectroscopic techniques

2-Hydroxy-5-methylbenzene-1, 3-dialdehyde, and 2-aminothiophenol were used as starting materials in the synthesis of the ligand. The solvents used in this project, which include ethanol, acetonitrile, and N,N dimethylformamide, are of high purity and manufactured by Merck. Metal salts were also obtained from Sigma-Aldrich. The available IR spectrum was taken by a Jasco FT/IR-4200 instrument. NMR spectra were taken by a Bruker NMR spectrometer (400 MH). All frequencies are in units of 1 cm wave number. UV–Vis spectra were recorded using a Varian Eclipse 300 spectrophotometer**.** A quartz cell with a path length of 10 mm was used in all experiments.

The Mass spectrometry experiments were performed on a Thermo LXQ ESI-MS with an ion trap mass separator. A solution of synthesized ligand (NC) was made at 1 mg mL^−1^ in LC/MS grade CH_3_CN. After a blank was recorded, the solution containing a ligand was injected into the instrument, and the mass spectra were recorded. The Mass spectrum of the chemosensor was reported in positive ion mode.

## 3 Results and discussion

### 3.1 FT-IR analysis

**Fig. 2:**
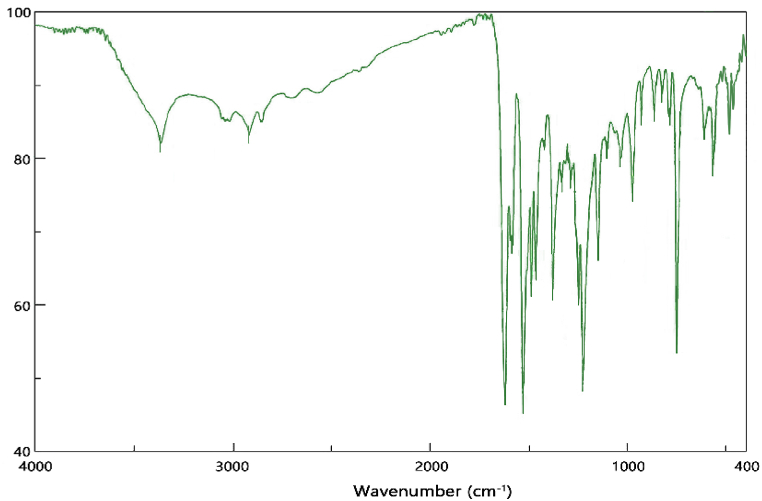
FT-IR spectrum of synthesized ligand.

The room temperature FT-IR spectrum of the synthesized ligand is shown in Fig. 2. No stretching vibration of the carbonyl (C=O) group is observed in this spectrum. In contrast, the stretching vibration of group C=N at 1623 cm^−1^ has appeared as a strong band. The band related to the stretching vibration of aliphatic protons appeared at 2857 cm^−1^ and 2917 cm^−1^ frequencies. The stretching vibration of aromatic protons was observed at 3040 cm^−1^ frequency, and the band related to the aromatic C=C group was observed at 1485 cm^−1^ frequency. The absence of both bands related to carbonyl and NH_2_ groups indicates that the Schiff base formation reaction has been completed.

### 3.2 ^1^H NMR analysis

**Fig. 3:**
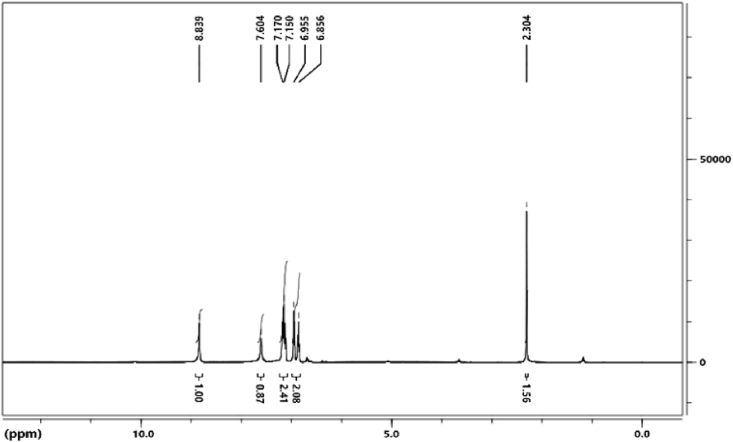
^1^H NMR spectrum for ligand.

The ^1^H NMR spectrum of the ligand is depicted in Fig. 3. In this spectrum, the single peak at 8.83 ppm is related to imine hydrogen. Five peaks in the range of 6.85–7.6 ppm indicate aromatic hydrogens, and one peak at 2.3 ppm indicates hydrogen related to the methyl group.

### 3.3 ^13^C NMR and mass spectrometry results

**Fig. 4:**
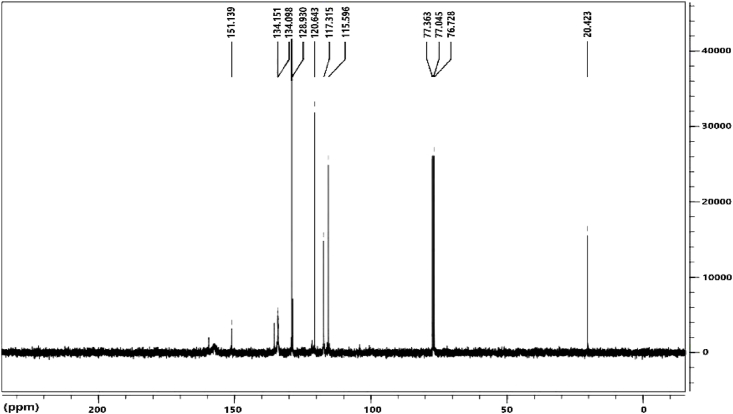
^13^C NMR spectrum for ligand.

**Fig. 5:**
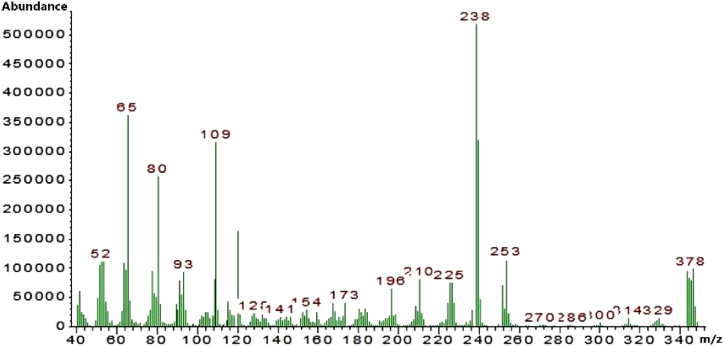
Mass spectrometry for ligand.

Fig. 4 shows the ^13^C NMR spectrum of the synthesized ligand. In this spectrum, the carbon corresponding to Schiff base appeared as a singlet at 151.13 ppm. Peaks in the range of 111.59–134.15 ppm indicate aromatic carbons. The single peak at 20.43 ppm corresponds to the carbon of the methyl group. Mass spectrometry analyses allow for the study of complex formation without the interference of solvents. As a result, the molecular mass of 378 *m*/*z* was calculated, which is related to the synthesized ligand (Fig. 5).

### 3.4 Optical properties

**Fig. 6:**
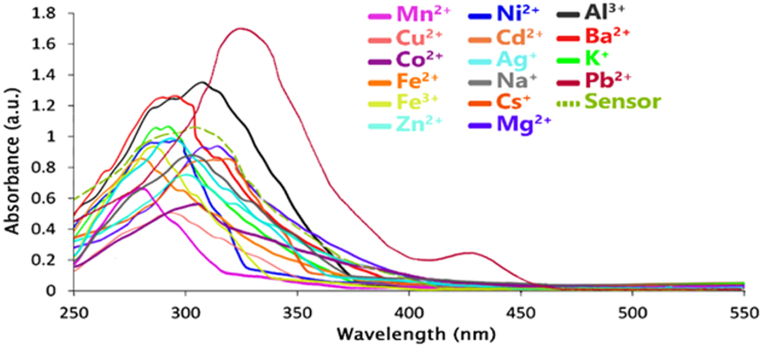
The UV–Vis absorption spectrum of the ligand in the presence of different cations.

The UV–Vis absorption spectra of the ligand in the absence and presence of various ions are shown in Fig. 6. It can be seen that the isolated ligand displays an absorption maximum $\left(\lambda_{\max}^{\text{Abs}}\right)\;\text{near}\;a$ 300 nm wavelength (see curve with dashed line). The response of the ligand to the presence of Pb^2+^ cations was more impressive than that of other ions. Therefore, with the addition of Pb^2+^ cations, the maximum absorption wavelength of the ligand changed from 300 nm to 325 nm. A new absorption band was formed at the wavelength of 430 nm. Meanwhile, with the addition of other ions, no noticeable change in the wavelength or intensity of ligand absorption was observed. The change in the absorption wavelength (with the addition of Pb^2+^ cations) is related to π→π* electron transitions. In the π to π* transition (especially in a polar solvent), the π* energy level will become more stable than the π energy level. For this reason, the energy level of π* will be higher than π. As a result, the distance between energy levels is reduced, and less energy is required for electron transfers. In this case, the absorption peak shifts to longer wavelengths. In the computational section, this issue has been addressed as much as possible by examining the HOMO-LUMO energy levels and the energy gap between them using density functional theory (DFT).

After the investigation of the spectral features of the sensor in the presence of Pb^2+^, solutions with the same concentration (4 × 10^−5^ M) of the sensor were prepared in ethanol solvent. The titration of these solutions was done using the standard solution of Pb^2+^ ions (0–6 equivalent). After the titration of the sensor solution with different concentrations of Pb^2+^, the absorption intensity of the band formed at 325 nm decreased, and a strong absorption band (caused by charge transfer) was formed at 430 nm.

**Fig. 7:**
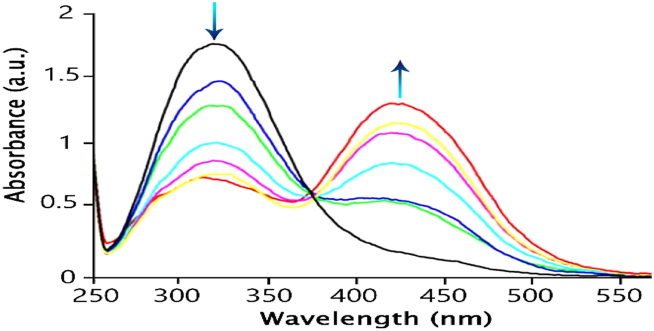
The UV–Vis spectrum of ligand titration against different concentrations of Pb^2+^ in an ethanol-water solution (v/v, 90,10).

The reason for this can be related to the interaction between Pb^2+^ ions and the OH-functional group. It seems that the negative charge produced by the deprotonation of the ligand by Pb^2+^ leads to a change in the wavelength and a change in the color of the solution (Fig. 7). On the other hand, the optical absorption spectra showed an easily detectable isobestic point at 375 nm, indicating the formation of a single discrete metal-ligand complex.

### 3.5 Colorimetric studies and naked-eye detection

**Fig. 8:**
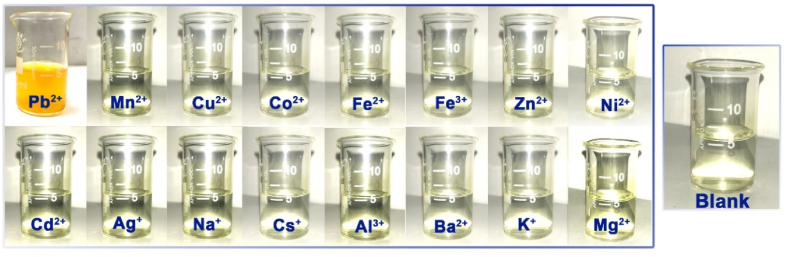
Sensor color change in the presence of different cations in an ethanol-water solution (v/v, 90, 10).

Today, the use of colorimetric sensors for the detection of important chemical species has expanded. The most important feature of this category of sensors is the identification of cations and anions through the unaided eye and without the need for an expert user. In this section, colorimetric studies were conducted to demonstrate the sensor responses of ligand in the presence of various metal ions (Mn^2+^, Cu^2+^, Co^2+^, Fe^2+^, Fe^3+^, Zn^2+^, Ni^2+^, Cd^2+^, Ag^+^, Na^+^, Cs^+^, Mg^2+^, Al^3+^, Ba^2+^, K+, and Pb^2+^) with the naked eye. For this purpose, a 15 μM solution of the sensor was prepared in distilled water. Identical sample vials were filled with 1 mL of sensor solution. One of the vials was kept pure. Then, 4 equivalents of Mn^2+^, Cu^2+^, Co^2+^, Fe^2+^, Fe^3+^, Zn^2+^, Ni^2+^, Cd^2+^, Ag^+^, Na^+^, Cs^+^, Mg^2+^, Al^3+^, Ba^2+^, K^+^, and Pb^2+^ ions were added to other vials. It is shown that the solution changed from colorless to purple with the addition of Pb^2+^ ions (See Fig. 8). This color change confirms the high selectivity of the sensor towards the presence of Pb^2+^ ions. This result may be caused by ligand-to-metal charge transfer (LMCT).

### 3.6 Limit of detection (LOD) of Pb^2+^ cation

The LOD parameter is calculated as the lowest concentration of an analyte in a sample using the equation LOD = 3σ/k [29,30]. In this relationship, k is the slope of the line curve, and σ is the standard deviation. A calibration curve was drawn based on the intensity of UV–Vis absorption at 430 nm in the range of 0–10 equivalents to evaluate the sensitivity of the synthesized ligand to the presence of Pb^2+^.

**Fig. 9:**
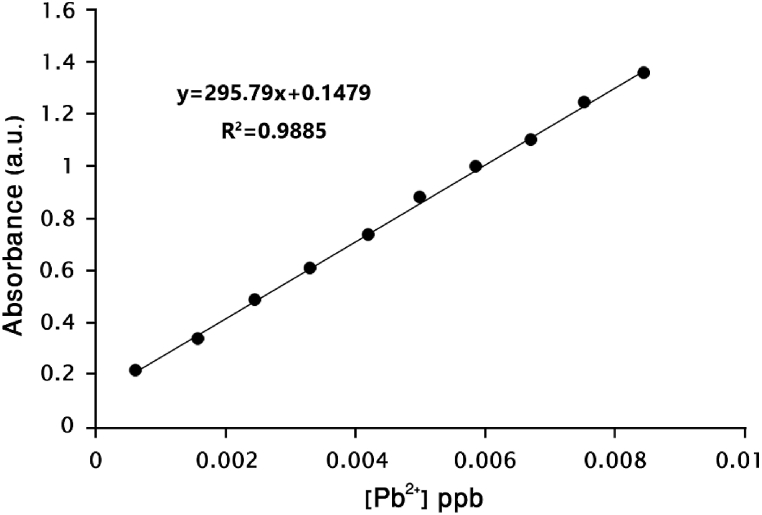
Sensor calibration curve in the presence of Pb^2+^ cations in an ethanol solvent.

The synthesized ligand can detect Pb^2+^ ions at a detection limit of 0.001 ppb (0.001 μg/liter), as calculated. This limit is significantly below the emission limit set by the World Health Organization (WHO-1984), indicating the sensitivity of the ligand towards Pb^2+^ ions. (Fig. 9).

**Fig. 10:**
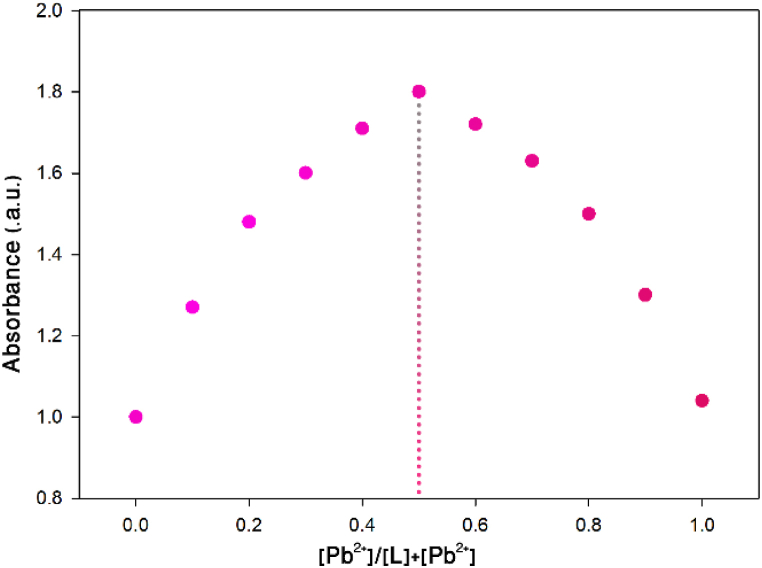
Job's plot for the Ligand/Pb^2+^ complex.

Also, according to the results of Job's plot which determines the stoichiometric ratio of the ligand/Pb^2+^ complex (Fig. 10), the maximum point is reached at a molar fraction of 0.5, indicating the formation of a host-guest complex with a ratio of 1:1 between the ligand and the Pb^2+^ cation.

According to the obtained results (very low LOD, high sensitivity to the presence of Pb^2+^, and no use of expert force), it seems that the synthesized ligand in this study has a high efficiency for the detection of Pb^2+^ ions in aqueous solutions. In fact, the synthesized ligand acts as a sensor to identify Pb^2+^, so that as soon as Pb^2+^ is present in the environment, it transfers the received chemical signal to the action potential and finally produces a detectable signal to confirm the presence of Pb^2+^. Table 1 shows a comparison of the sensors described in the literature with those in our compound.

**Table 1:** Selectivity comparison between some studied compounds from the literature and our newly investigated ligand.

Literature	LOD (ppb)
[31]	0.0206
[32]	0.3
[33]	1
[34]	0.15
[35]	0.01
**The sensor studied in this work**	0.001

### 3.7 Theoretical/quantum section

In this part of the work, theoretical calculations were used to support the measurements. In this regard, DFT and QTAIM methods have been applied. For this purpose, first, the structure of the ligand in the presence or absence of Pb^2+^ was optimized using DFT theory at the theoretical level of B3LYP/6-311g+(d,p) in water solvent using the implicit conductor-like polarizable continuum model (CPCM). Then its electronic properties (i.e., the HOMO and LUMO energy levels, the energy gap, and the absorption energy) were investigated. The TD-CAM-B3LYP method was employed to simulate the UV–Vis optical spectra of the formed ligand-Pb^2+^ complexes. Furthermore, the QTAIM method was employed to study the characteristics of significant atomic regions.

**Fig. 11:**
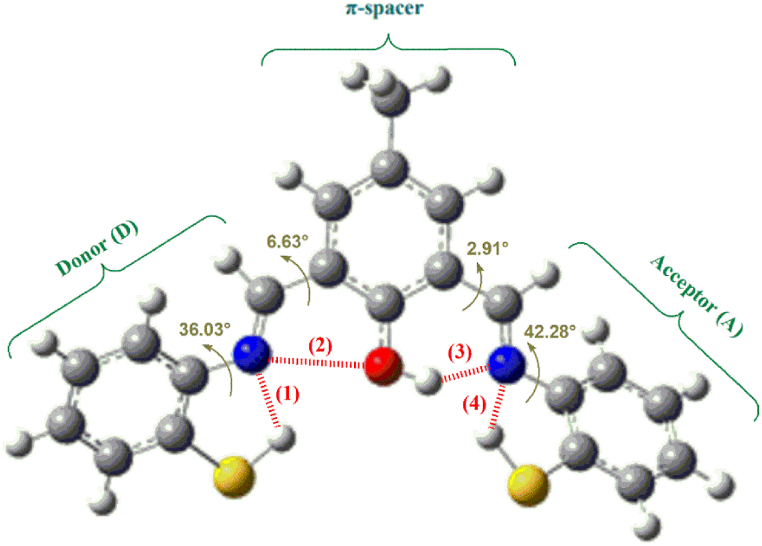
Non-planar molecular optimized structure of the studied ligand at the DFT//B3LYP/6-311g+(d,p), recorded in water. The intramolecular non-covalent interactions (NCIs) are indexed.

The ground (S_0_) state optimized structure of the studied ligand in water is illustrated in Fig. 11.

Fig. 11 displays two types of short contacts: N⋯H ((1), (2), and (3)) and N⋯O (2) at the optimized ground (S_0_) state [(1): 2.17 Å, (2): 2.80 Å, (3): 1.69 Å and (4): 2.38 Å]. All the short contacts, or NCIs, are much smaller than the usual sum of their corresponding VdW radii. We will return to the possibility of investigating the NCIs by using the Reduced Density Gradient (RDG) at low densities.

#### 3.7.1 Molecular electronic properties

##### 3.7.1.1 MEP map

**Fig. 12:**
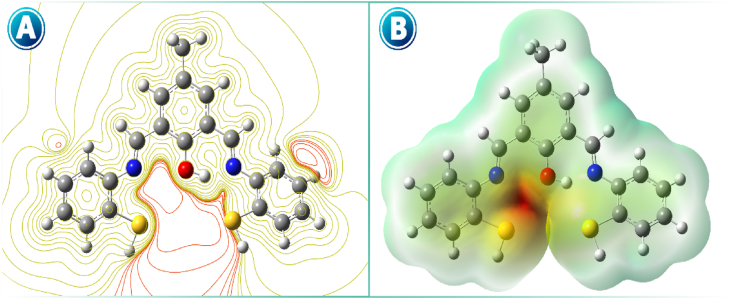
Contour map of electron density Laplacian (A) and MEP (B) of isolated-ligand.

MEPs provide comprehensive data for research on the chemical reactivity or biological activity of a compound. Electrophilic or nucleophilic attacks are principally determined by the MEP. Moreover, the active site of a receptor's substrate binding is mainly determined by the spatial arrangement of the electrostatic potential in three dimensions [36]. Contour MEP, the map, offers a simple tool to predict how different geometries can interact (see Fig. 12 (A)). The electrostatic potential map is investigated by the B3LYP-DFT/6–311+g(d,p) method and is shown in Fig. 12 (B). In the MEP map, the regions with the highest positive/negative charge are the preferred sites for electrophilic/nucleophilic attack and are denoted by the blue/red colors, respectively. The significance of MEP lies in its ability to display molecular size, shape, and electrostatic potential regions of positive, negative, and neutral charge simultaneously, using color grading, making it very useful in the study of molecular structure and its physiochemical property relationships. The electrostatic potential increases in the following order: red < orange < yellow < green < blue [37]. The region of red color (electron-rich) is located around the nitrogen and oxygen atoms, where electrophilic attack is possible. The yellow color region (electron-poor) is spread all over the molecule around hydrogen and carbon atoms, which is prone to nucleophilic attack [38].

It is clear that the oxygen and nitrogen atomic basins are surrounded by negative electrostatic potentials, and the MEP around these atoms is more extended than that around other atoms in space. This appears because of the high electronegativity of these atoms. Therefore, it seems that the atomic basins of oxygen and nitrogen are the active places of charge and energy exchange and the places of connection of Pb^2+^ in the ligand.

##### 3.7.1.2 Energy gap

The HOMO and LUMO energy levels are important because they indicate how a molecule interacts with other chemical species. The energy difference between the HOMO and the LUMO, known as the HOMO-LUMO energy gap, plays a vital role in the charge transfer interaction within the molecule and aids in predicting its electrical transport characteristics [39].

The DFT-based computed HOMO and LUMO energy values are −6.014 eV and −2.363 eV, respectively. The calculated band gap was estimated to be 3.651 eV. A compound with a large energy gap has lower chemical reactivity and better kinetic stability. Since the isolated ligand was non-centrosymmetric, it is clear from Fig. 11 that the compound exhibits a large dipole moment (9.134 Debye). The difference in the dipole moments (Δμ) between the ground and excited states is responsible for the different nonlinear properties [40].

Donor-acceptor (D-A) organic compounds are one of the crucial conjugated systems that generate a narrow energy band gap that is advantageous in various technological domains for developing innovative materials [41]. In this case, the transfer of electrons from the conduction band to the valence band is facilitated. As a result, reactivity and absorption energy increase.

**Fig. 13:**
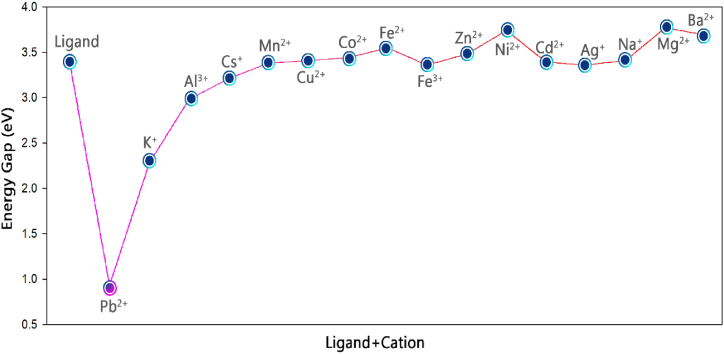
Energy gap changes in the presence of different cations.

The results show that the ligand energy gap was significantly reduced by adding Pb^2+^ (see Fig. 13). The reason for this is related to the excessive combination in π bonds and the quantum tunneling phenomenon in the ligand/cation system.

**Fig. 14:**
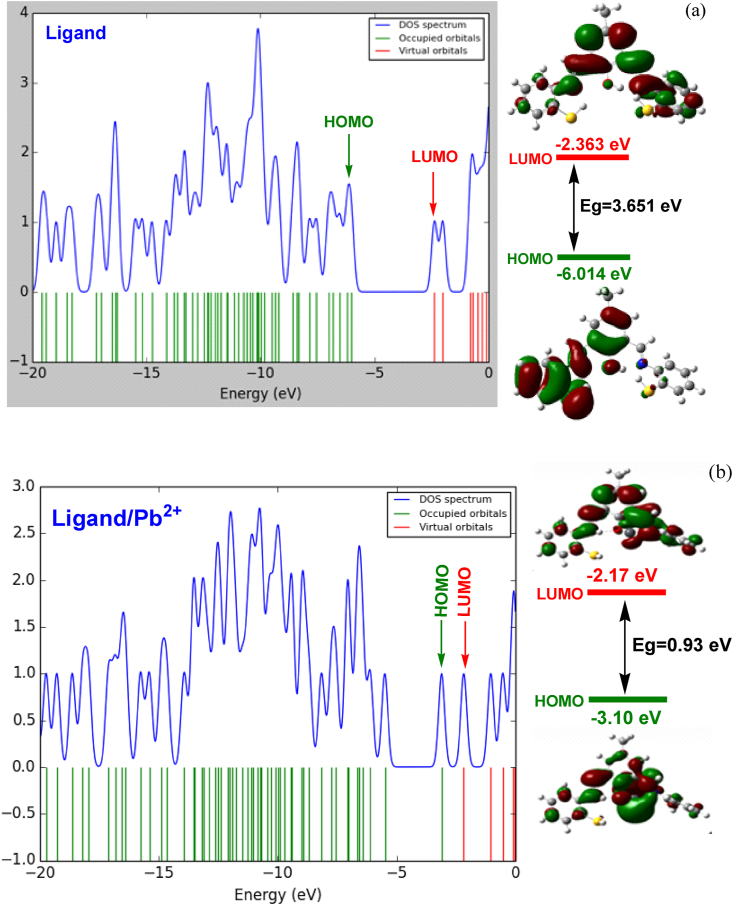
DOS spectra and FMO plots for ligand (a) and ligand/Pb^2+^ complex (b).

In addition, in order to investigate the electronic properties in the presence or absence of lead cations, the electron density of the state spectrum (DOS) was calculated for them using the GaussSum program (Fig. 14 (a) and (b)). The DOS diagram of a molecular system actually describes the number and energy of excited and unoccupied quantum states (molecular orbitals) in that system. The electronic structure of a material, such as its density of states (DOS), offers crucial insights into its physical and functional properties [42].

Based on the frontier molecular orbitals (FMOs) plot (Fig. 14 (a) and (b)), it is interesting to note that the donor (D)-π spacer-acceptor (A) framework with the electronic push-pull effect provides suitable molecular architecture for the molecular design of the isolated ligand. In addition, a comparison of the DOS plots of the ligand in the presence or absence of lead cations shows that the energy gap decreases significantly after the adsorption of the lead cation. Therefore, the ligand can act as a sensor for the Pb^2+^ cation.

##### 3.7.1.3 Adsorption energy

The adsorption mechanism is therefore founded on the relationship between the adsorbate and a surface, which can involve various processes such as covalent or ionic chemical bonds, van der Waals interactions, and dipolar interactions [43,44]. In this regard, DFT simulations have been conducted to investigate the energy of lead adsorption. The E_ads_ of the complex was evaluated based on Eq. (1).

(1) $$E_{\text{ads}}=E_{\text{Complex}}-\left(E_{\text{Cation}}+E_{\text{Ligand}}\right)$$

**Fig. 15:**
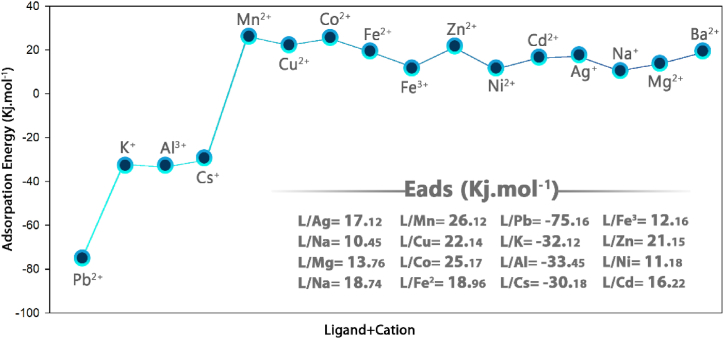
E_(ads)_ energy for Ligand/M^+^.

The calculated $E_{\text{ads}}$ value for the ligand/Pb^2+^ complex shows that the adsorption of Pb^2+^ on the ligand has a very good chemical nature. The decrease in the energy gap after the addition of Pb^2+^ is in agreement with the high adsorption energy between the ligand and Pb^2+^ (Fig. 15).

##### 3.7.1.4 Optical properties

**Fig. 16:**
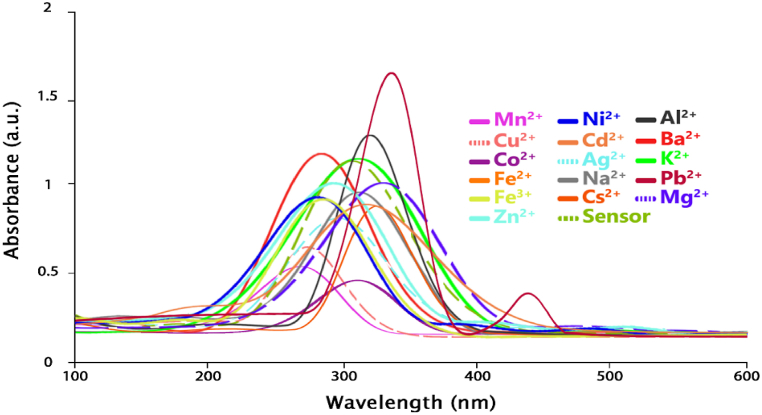
UV–Visible spectrum of ligand and ligand/M^+^ complexes.

In order to investigate the optical characteristics of the ligand and the complex formed by the ligand and Pb^2+^, the UV–visible spectra were computed using the TD-DFT approach with the CAM-B3LYP/6–311+g(d,p) level of theory in water. The simulated UV–Vis spectra are given in Fig. 16.

The maximum intensity of ligand adsorption is observed at 295 nm (without cation). The ligand-Pb^2+^ compound displays a noteworthy increase in both the absorption intensity and the wavelength maximum, in contrast to the existence of other cations, as can be easily discerned. Furthermore, a shoulder appears at 450 nm, which can be attributed to ICT as reported for donor-acceptor systems. These findings suggest that the ligand exhibits strong sensitivity and selectivity for Pb^2+^ through enhanced optical properties. Taking account of what has been said before; it is expected to measure Pb^2+^ in different water samples. These findings showed high agreement with the experimental results.

##### 3.7.1.5 QTAIM approach

**Fig. 17:**
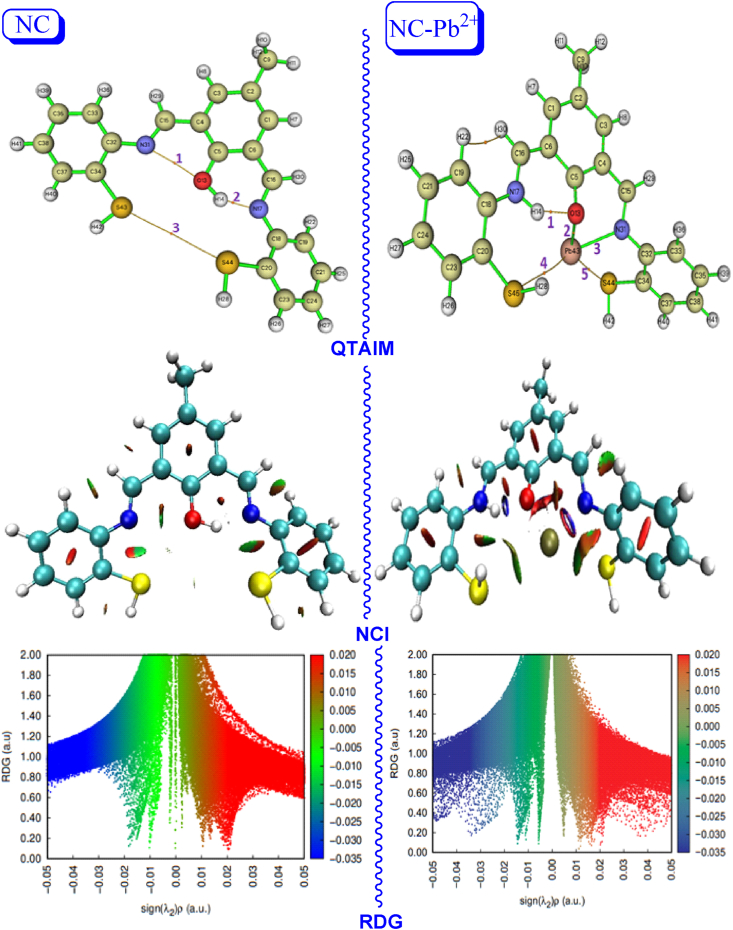
QTAIM, NCI, and RDG graphs of the NC and NC-Pb^2+^ complex.

In this section, the QTAIM was used to explore the chemical interaction between the ligand (NC) and the Pb^2+^ cation [[45], [46], [47], [48], [49]]. The QTAIM, as proposed by Bader [26], categorizes the various interactions within molecular systems and characterizes bonding interactions through real-space functions, like the electron density found at the bond critical points (BCPs) [50]. Based on QTAIM analysis, topological properties of electron density and the Laplacian are employed to elucidate interatomic interactions. Also, based on the morphology of electron density (ρ (r)) and its Laplace (∇^2^ρ (r)), the potential energy density (V(r)) and the kinetic energy density (G(r))of molecular structures can be calculated. Also, the interaction energy (E_int._) is calculated using the equation of Espinosa et al., **as** E_int_ V(r)/2 [51]. The QTAIM topological parameters are shown in Table 2. The AIM graphs and NCI-RDG plots are depicted in Fig. 17. After the addition of the Pb^2+^ cation, the appearance of the three intra-molecular bonds is noted, which are responsible for stabilizing the NC system. The first is built between the atoms O13 and N31. This interaction is characterized by an electron density of order 0.0151 a.u. The Laplacian is equal to 0.0659. The |V(r)|/G(r) ratio is outside the rule of Bianchi et al. (1<|V(r)|/G(r) < 2) [52]. This justifies the fact that the interaction is closed. This finding is confirmed by the appearance of the red spot between the oxygen and nitrogen atoms (see NCI graphs). This BCP1 is characterized by an interaction energy (Eint.) of 20.21 kJ mol^−1^.

**Table 2:** The QTAIM topological parameters (in a.u.) of the NC and NC-Pb^2+^: electron density ρ(r), laplacian of electron density ∇^2^ρ(r), Lagrangian kinetic energy density G(r), potential energy density V(r), |V(r)|/G(r) ratio, and the interaction energy E_int_ in kJ.mol^−1^ at the selected BCPs.

Compounds	BCPs	ρ(r)	∇^2^ρ(r)	G(r)	V(r)	|V(r)|/G(r)	E_int._
NC	BCP1	0.0151	0.0659	0.0159	−0.0154	0.96	20.21
	BCP2	0.0721	0.1062	0.0498	−0.0732	1.46	96.09
NC-Pb^2+^	BCP1	0.0571	0.1120	0.0484	−0.0689	1.42	90.31
	BCP2	0.0874	0.2882	0.0400	−0.0615	1.53	80.60
	BCP3	0.0654	0.0365	0.0248	−0.0365	1.47	47.78
	BCP4	0.0056	0.0123	0.0024	−0.0020	0.83	12.62
	BCP5	0.0147	0.0380	0.0068	−0.0066	0.97	8.66

In BCP2, the value of ρ(r) is 0.0721 a.u., and its ∇^2^ρ(r) is equal to 0.1062 a.u. It is obtained that the |V(r)|/G(r) ratio is in the interval of Bianchi et al. (1 <|V(r)|/G(r) = 1.46 < 2). This idea deduces that the interaction formed between H14 and N17 is a covalent bond. From the NCI idex, a blue spot is obtained between the two atoms, which deduces that the interaction is covalent. The E_int._ energy is around 96 kJ mol^−1^. Also, a low level of interaction is formed between the two S atoms, balancing the two symmetrical groups (Fig. 17). The addition of a Pb2+ cation in the active region disrupts the NC system, forming four bonds with it. Also, the NC compound forms an intra-molecular hydrogen bonding interaction for stabilized H14–O13. From Tables 2 and it is found that this interaction is characterized by an energy of 90.31 kJ mol^−1^. The electrons given up by the Pb2+ cation are attracted by the oxygen, nitrogen, and sulfur atoms, forming with them two hydrogen and two van der Waals interactions. In Fig. 17, the NCI plots show that the two H-bonding interactions are Pb⋯N (BCP2) and Pb⋯O (BCP3). It is observed that the van der Waals interactions are Pb⋯S45 and Pb⋯S44 (see NCI index). These H-bonding interactions are responsible for capturing Pb^2+^ by the NC molecule. It is shown that these interactions have energy binding values equal to 80.60 kJ mol^−1^ and 47.78 kJ mol^−1^, respectively. In BC2 and BCP3, the electron density ranges from 0.0874 a.u. to 0.0654 a.u. The Laplacian of electron density is in the range of 0.2882–0.0365 a.u. These positive values are an indication that the interaction is covalent. It is clearly shown that the |V(r)|/G(r) ratio is around 1.5 a.u. This value is lower than the unit. Therefore, it is concluded that the interaction is covalent. This finding confirms the QTAIM topological analysis. The RDG graph (Fig. 17) shows two sharp peaks directed towards 0.010 a.u. and 0.015 a.u. This finding confirmed our results that Pb^2+^ forms two hydrogen-bonding interactions that stabilize it in each active region. Therefore, the NC is highly selective for the Pb^2+^ cation.

The QTAIM topological parameters and the NCI-RDG plots have demonstrated that Pb^2+^ is well stabilized in its active region via van der Waals and H-bonding interactions. Therefore, the NC material is well selective and reactive to the Pb^2+^ cation.

##### 3.7.1.6 IGM/ELMO analyses

**Fig. 18:**
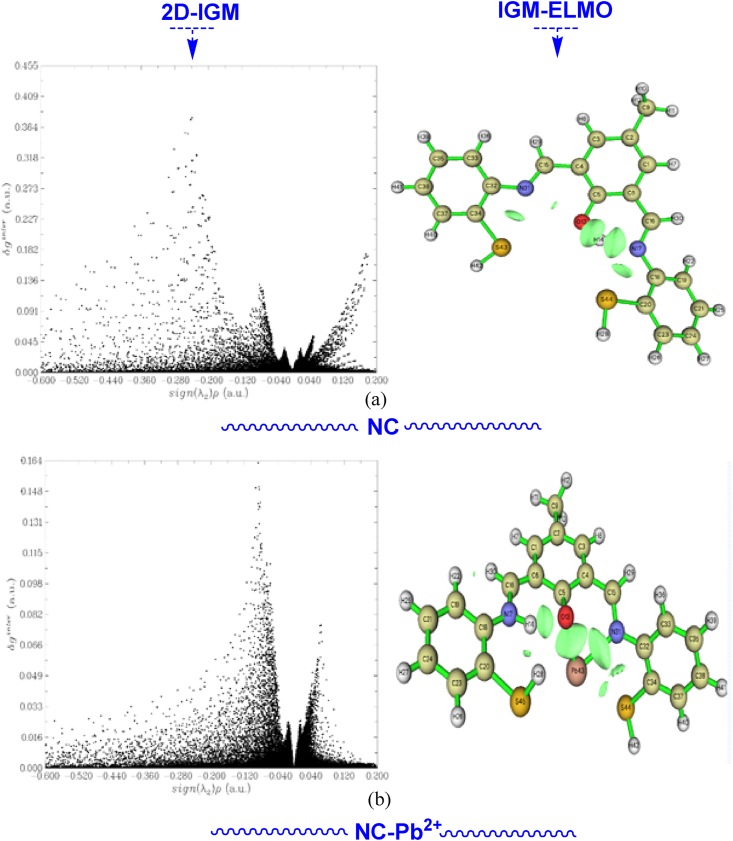
IGM-ELMOs plots of NC (a) and NC-Pb^2+^ complex (b) δg^inter^. (isovalue = 0.002).

The experimental studies, electronic properties, and QTAIM-RDG analyses have clearly demonstrated that the NC compound is very suitable to capture the Pb^2+^ cation. Therefore, to confirm this finding and to demonstrate that the NC is very selective to Pb^2+^ via hydrogen bonding interactions, we have focused on the Independent Gradient Model (IGM) and the extremely localized molecular orbitals (ELMO) analyses [[53], [54], [55]]. The IGM theory is based on the δg^intra/inters^ parameters, which are the key functions of this theory. It has been demonstrated which interactions are responsible for the capitation of the Pb^2+^ cation. In Fig. 18 (a) and (b), the δg^inter^ is represented as a function of sign(λ2).ρ. Also, the ELMO is an efficient method to show the electronic orbital accumulation in the active area. Therefore, it is a powerful method to visualize the electrons that are responsible for the formation of hydrogen bonding interactions between the NC and Pb^2+^ cations. An IGM-ELMO calculation is very beneficial to confirm the presence of the hydrogen bonding interactions forming between NC and the Pb^2+^ cation. The 2D-IGM and IGM-ELMO are depicted in Fig. 18. In NC, from 2D-IGM, three solid peaks are observed located around −0.40, 0.40, and 0.035 a.u. These stronger peaks indicate the stabilization of the NC compound via intra-molecular hydrogen bonding interactions. In addition, it appears there are three diffusive peaks at about −0.090, −0.240, and 0.180 a.u. This finding demonstrated the formation of intramolecular van der Waals interactions. These results are well confirmed by the QTAIM and NCI-RDG analyses. From Fig. 18 (a), the IGM-ELMO obtained the accumulation of electronic orbitals in the more active sites. Therefore, this region is a very select one for the detection of the Pb^2+^ cation. Concerning the NC-Pb^2+^, it is clearly shown from 2D-IGM that there are two solid peaks and two diffusive peaks. The solid peaks are about −0.040 a.u. and 0.040 a.u., and the diffusive peaks are about −0.090 a.u. and 0.033 a.u., respectively. This finding concluded the formation of two hydrogen bonds and two van der Waals interactions between the NC and Pb^2+^ cations. Consequently, this result confirmed our QTAIM analyses. It is concluded that Pb^2+^ is well selected by the NC via hydrogen bonding interactions. From IGM-ELMO (see Fig. 18 (b)), it is found that there is an accumulation of electronic orbitals in excess between Pb^2+^ and NC. It is deduced that charge transfer occurs between the two systems. This idea concluded the possible formation of hydrogen bonding interactions between the NC and Pb^2+^. Moreover, it is clearly shown that the highly electronic accumulation is found between Pb and N, and Pb and O. This idea implies that Pb^2+^ is fixed in each active region via hydrogen bonding interactions.

A correlation between IGM and ELMOs demonstrated that the Pb2+ cation is attacked by hydrogen bonds to be well stabilized. Otherwise, NC is a promoter compound for detecting Pb2+.

##### 3.7.1.7 Electron localization function (ELF) analysis

Surface analysis of the NC and NC-Pb^2+^ materials is dependent on the repartition of electron bond critical points (BCP) in the active region and is investigated by ELF and LOL theories. The highly active regions in the presence of the Pb^2+^ cation are investigated. This finding refers to the presence of the electron lone pair on the molecular surface, which facilitates the stabilization of the selected cation via hydrogen bonding interactions. The electron localization function (ELF) is an efficient theory to show the electron occupation sites after sensing cations [[56], [57], [58]]. It is indicated that a donor-acceptor couple exists between the selected cation and the NC molecule. As a result, the NC's sensitivity to Pb^2+^ cations has been confirmed.

**Fig. 19:**
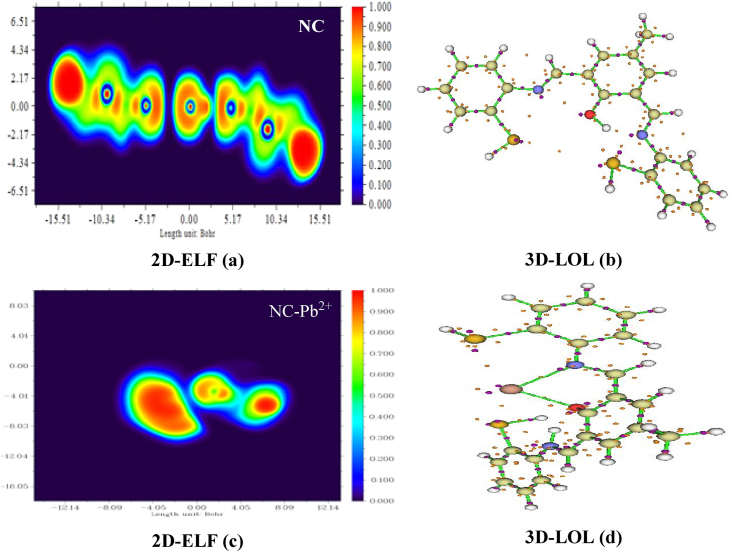
2D-electron localization function (ELF) maps and 3D localized locator (LOL) graphs of the studied compounds (NC: (a) and (b), NC-Pb^2+^: (c) and (d)).

The ELF theory is elaborated by Becke and Edgecombe [59]. The ELF is computed along the (X, Z) plane. The 2D-ELF and 3D-LOL graphs are depicted in Fig. 19(a–d). From 2D-ELF Fig. 19 (a), it is shown that red spots appeared in the region of interaction. This finding deduces the presence of a maximum accumulation of electrons in this region. Then, it is justified to expect the possible formation of hydrogen bonding interactions with the Pb^2+^ cation. In addition, it is concluded that the possible charge phenomena are taken into account between NC and Pb. From 3D-LOL maps (see Fig. 19 (b)), it is indicated that there are large shark stains around the O, N, and S atoms. Therefore, it is a sign that the NC molecule is stabilized by intra-molecular hydrogen and van der Waals interactions. These results confirmed our QTAIM analyses. Concerning the NC-Pb^2+^ complex, it is clearly shown that a large red spot appeared in the interaction site (see 2D-ELF, Fig. 19 (c)). This idea explains the presence of an excess of isolated lone pairs of atoms around the neighboring Pb^2+^ cation. So, it is concluded that charge transfer occurs between NC and Pb^2+^. It is an indication of the stability of Pb due to hydrogen bonding interactions. This idea is well confirmed by 3D-LOL plots (see Fig. 19 (d)); it is clearly shown that the Pb^2+^ is fixed in its active region by two hydrogen bonding interactions (Pb⋯O and Pb⋯N). Also, this interpretation well confirms our QTAIM results. It is deduced that NC is highly selective and reactive to the Pb^2+^ cation. It is determined that the ELF value ranges from 0.85 a.u. to 1 a.u. Finally, ELF-LOL analyses confirm that Pb^2+^ is well stabilized in its active region via hydrogen bonding interactions. Therefore, the NC is suitable for selecting a Pb^2+^ cation.

## 4 Conclusion

In this paper, a new chemosensor for the detection of Pb^2+^ was investigated via DFT and experiment. The synthesized sensor was confirmed through ^1^H and ^13^C NMR, FT-IR, and Mass spectrometry analyses. According to the WHO limits for the presence of lead in water environments, the selective detection of lead ions seems necessary. Thus, this study focused on the selective detection of Pb^2+^. The obtained results showed that the synthesized ligand is very suitable to detect Pb^2+^ cations (limit of detection (LOD)) up to 0.001 ppb (0.001 μg/M). The experimental characterizations showed that the ligand is based on a 2-hydroxy-5-methylbenzene-1,3-dialdehyde conjugated with an electron-donating group (2-aminothiophenol), which provides strong binding to the Pb^2+^ cation. The linker and electron acceptor enable long-wavelength intrinsic charge transfer (ICT) optical absorption, which is desirable for sensor development. The yellow color observed in the ligand-Pb^2+^ complex is a result of the π→π* electronic transition. To validate the experimental findings, theoretical calculations were conducted using DFT and QTAIM methods. The acquired results highlighted that all the parameters, including adsorption energy, wavelength, and charge/energy transfer rate, considerably improved in the presence of Pb^2+^.

We conclude that the synthesized ligand can be used as a novel chemical sensor due to its excellent sensitivity and selectivity, short detection time, no need for skilled labor, easy and cheap synthesis, and very low detection limit (LOD). Consequently, we anticipate that the findings of this investigation will establish a novel class of efficient chemical sensors for the detection of Pb^2+^. Finally, this powerful new material for the detection of lead is a new concept that can be used in many applications (Environmental, biological, and pharmaceutical).

## Author contribution statement

Hamid Hadi: Gassoumi Bouzid: Conceived and designed the experiments; Performed the experiments; Analyzed and interpreted the data; Contributed reagents, materials, analysis tools or data; Wrote the paper.

Samia Nasr: Houcine Houcine Ghalla: Rafik Ben Chaabane: Analyzed and interpreted the data.

Sahbi AYACHI: Analyzed and interpreted the data; Wrote the paper.

## Data availability statement

Data included in article/supp. material/referenced in article.

## Additional information

No additional information is available for this paper.

## Declaration of competing interest

The authors declare that they have no known competing financial interests or personal relationships that could have appeared to influence the work reported in this paper.
